# Biosynthesis and Antimicrobial Activity of Semiconductor Nanoparticles against Oral Pathogens

**DOI:** 10.1155/2014/347167

**Published:** 2014-03-04

**Authors:** C. Malarkodi, S. Rajeshkumar, K. Paulkumar, M. Vanaja, G. Gnanajobitha, G. Annadurai

**Affiliations:** Environmental Nanotechnology Division, Sri Paramakalyani Centre for Environmental Sciences, Manonmaniam Sundaranar University, Alwarkurichi, Tamilnadu 627412, India

## Abstract

Dental care is an essential phenomenon in human health. Oral pathogens can cause severe break which may show the way to serious issues in human disease like blood circulation and coronary disease. In the current study, we demonstrated the synthesis and antimicrobial activity of cadmium sulphide and zinc sulphide nanoparticles against oral pathogens. The process for the synthesis of cadmium sulphide (CdS) and zinc sulphide (ZnS) nanoparticles is fast, novel, and ecofriendly. Formation of cadmium sulphide (CdS) and zinc sulphide (ZnS) nanoparticles was confirmed by surface plasmon spectra using UV-Vis spectrophotometer. The morphology of crystalline phase of nanoparticles was determined from transmission electron microscopy (TEM) and X-ray diffraction (XRD) spectra. The average size of cadmium sulphide (CdS) and zinc sulphide (ZnS) nanoparticles was in the range of 10 nm to 25 nm and 65 nm, respectively, and the observed morphology was spherical. The results indicated that the proteins, which contain amine groups, played a reducing and controlling responsibility during the formation of cadmium sulphide (CdS) and zinc sulphide (ZnS) nanoparticles in the colloidal solution. The antimicrobial activity was assessed against oral pathogens such as *Streptococcus *sp. *Staphylococcus *sp. *Lactobacillus *sp., and *Candida albicans *and these results confirmed that the sulphide nanoparticles are exhibiting good bactericidal activity.

## 1. Introduction

Dental caries and periodontal disease, the majority widely increased disease affecting mankind, occupy the devotion of microbes and expansion of biofilm on the natural and restored tooth surface equally. In this framework, a biofilm can be classed as an aggregate of bacteria in which cells stay to each other and to an outside [1]. Nanostructured materials are a technically significant object that possesses optical and electrical properties that depend powerfully on the size and shape of the nanoparticles. This is due to confinement of the charge carriers in the narrow space of the nanocrystal [2, 3]. Semiconductor and other nanoparticles are currently being combined by polymers or coated onto surfaces which may have a multiplicity of potential antimicrobial applications with the oral cavity [4, 5]. Recently, II-VI semiconductor nanoparticles are playing attention in enormous fields due to their excellent and unique optical and electrical properties which present a major advantage over their mass counterparts [6–8]. Polymers are also excellent host materials as capping agents and stabilizers since they prevent agglomeration and precipitation of the particles. Sulfide is a semiconductor nanomaterial processing a lot of interesting physical properties and potentially used in mesoscopic electronic [9] biolabeling [10] and photocatalysis [11]. Metals have been used for centuries as bactericidal agents; silver, copper, gold, titanium, and zinc have attracted particular attention, each having various properties and spectra of activity [12].

Various oral foods, including toothpaste, now integrate powdered zinc citrate or acetate to control the development of dental plaque [13]. Powdered titanium oxide is also generally used as a whitener in toothpastes [14]. The antibacterial, antifungal, and antiviral actions of sulfide nanoparticles have been broadly investigated in comparison with other metals. The use of silver nanoparticles has been well thought of for a range of biomedical applications, including, within the dental field, an antibacterial factor in dental resin composites [15]. The anticipation of dental caries and periodontal infection is usually targeted at automatic or nonspecific control of the plaque biofilm; biofilms are part of our daily life, for example, when brushing our teeth [16]. The use of bactericidal agents represents an expensive balance to mechanical plaque control [17]. However, real periods of exposure to antimicrobial agents through tooth brushing and mouth rinsing can be present especially short, amounting to 30 seconds [18].

In this work, we report extracellular synthesis of cadmium sulphide and zinc sulphide nanoparticles by the bacteria,* K. pneumoniae*, as reducing agent and find the effective factors for synthesis process. UV-Vis spectrophotometer was used to characterize the synthesized sulphide nanoparticles.

## 2. Materials and Methods

### 2.1. Isolation and Identification

The sulphur reducing bacteria were isolated from salt pan soil collected from Tuticorin. The collected samples were inoculated into a specific mineral medium. The isolated organism was maintained in sulphur reducing medium. The organisms were cultivated in 1 liter of specific mineral medium containing 1.5 g of sodium sulphate, 0.5 g of di-potassium hydrogen phosphate, 3.5 g of sodium lactate, 1.0 g of beef extract, 2.0 g of peptone, 0.1 g of calcium chloride, 0.392 g of Ferrous ammonium sulphate, 2.0 g of magnesium sulphate, and 0.1 g of sodium ascorbate. The organism was incubated at 30°C to 35°C. The isolates were morphologically and microbiologically characterized as* Klebsiella pneumonia* (strain MAA). Further,* Klebsiella pneumoniae* was maintained by subculturing in nutrient agar plates for production of cadmium sulphide and zinc sulphide nanoparticles.

### 2.2. Extracellular Synthesis of CdS and ZnS Nanoparticle

In this present study, we synthesized sulfur-based semiconductor nanoparticles such as CdS and ZnS, respectively. For CdS and ZnS nanoparticle synthesis, we have taken the precursor compounds like cadmium sulphate (CdSO_4_) and zinc sulphate (ZnSO_4_) at a concentration of 1 mM in 250 mL Erlenmeyer flasks. The bacterial supernatant,* Klebsiella pneumoniae* (MAA), was obtained from sterile conical flask and 1 mM of cadmium sulphate and zinc sulphate was mixed and the solution was incubated at 35°C for 24 hrs. After incubation of the above mixture, the preliminary detection of nanoparticles synthesis was carried out by visual observation of color change of the biomass. The reaction between the supernatant and CdS and ZnS ions was carried out in bright conditions for 24 hrs. Periodically, the synthesis of the CdS and ZnS ions in the solution was monitored by UV spectrophotometer at different time intervals. It can give more information about the shape and structure of the particles.

### 2.3. Characterization of CdS and ZnS Nanoparticles

The synthesis of CdS and ZnS ions in aqueous solution was monitored by UV-Vis spectrophotometer of the solution between 300 and 600 nm using PerkinElmer spectrophotometer. The nanoparticles were scanned by the infrared in the region of ~400–2500 cm^−1^ Fourier Transform Infrared spectrometer (Thermo Nicolet Model-6700). The CdS and ZnS nanoparticle suspension was air-dried on the specimen grid and was observed with a JEOL JEM-1010 Scanning Electron Microscope. The crystalline phases of the products were determined by X-ray powder diffractometer (Seifert-3000p). For Energy Dispersive X-ray analysis, the dried CdS and ZnS nanoparticles was placed on a carbon coated copper grid and performed on a HITACHI SU6600 model.

### 2.4. Antimicrobial Activity of Sulphide Nanoparticles

The well diffusion method was used to study the antibacterial activity of the synthesized CdS and ZnS nanoparticles. All the glassware, media, and reagents were used to sterilize an autoclave at 121°C for 20 minutes. The pathogenic bacteria,* Streptococcus* sp.,* Staphylococcus aureus, Lactobacillus* sp., and* Candida albicans*, were used as model test strains. The pure bacterial cultures were subcultured on nutrient broth medium. Each strain was swabbed uniformly onto the individual Muller Hinton agar plates using sterile swabs. Well of 6 mm diameter was made on Muller Hinton plates using gel puncture. Using a micropipette, the different concentration like 100**** *μ*L, 200 *μ*L, and 300 *μ*L of CdS and ZnS nanoparticles solution was poured onto each well on all plates and incubated at 37°C for 24 hrs. After incubation, the different levels of zone of inhibition (ZOI) of bacteria were measured.

## 3. Result and Discussion

### 3.1. Isolation and Identification

The bacterial strain is used for synthesis of CdS and ZnS nanoparticles which was isolated from saltpan soil from Tuticorin. The isolated strain MAA was morphologically and biochemically identified as* K. pneumoniae*.* K. pneumoniae* MAA was a gram negative, rod shaped, and nonmotile bacterium and was maintained at Microbial Type Culture Collection and Gene bank (MTCC), Chandigarh.

### 3.2. Synthesis of Sulphide Nanoparticles

#### 3.2.1. Optical Observation

**Figure 1:**
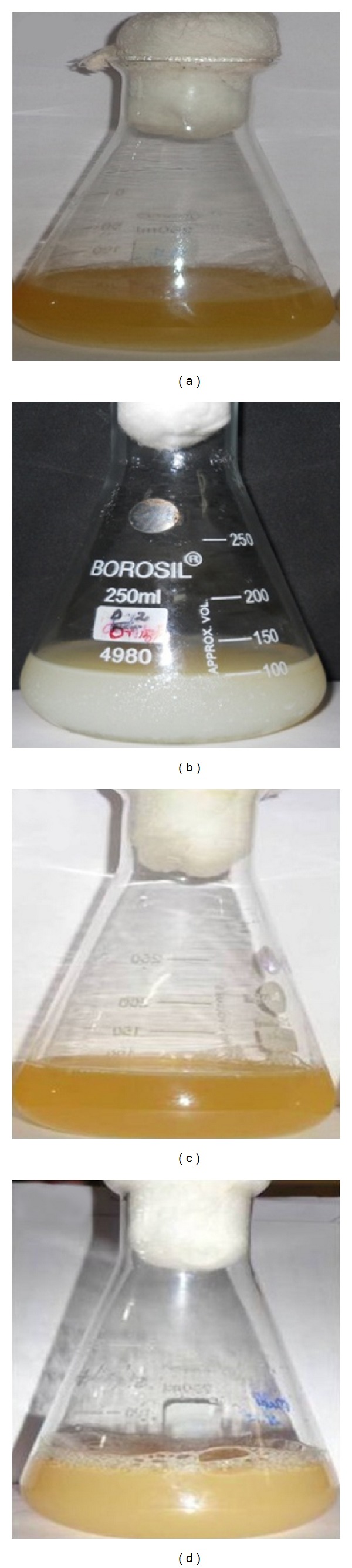
Biosynthesis of CdS and ZnS nanoparticles: (a) and (c) culture supernatant, (b) and (d) with addition of metal ions 24-hour incubation.

The present study shows the synthesis of CdS and ZnS nanoparticles by using the bacteria,* K. pneumoniae*. Herein, the sulphate was reduced into sulphide nanoparticles. After 24-hour incubation of CdSO_4_ with* K. pneumoniae* biomass, the formation of white color reveals the synthesis of CdS nanoparticles; after 24 hrs, the precipitation of CdS NPs on the absorbance bottom of the conical flask indicates that the CdS NPs synthesis process was completed (Figures 1(a) and 1(b)). The decreased absorbance of CdS nanoparticles in UV-Vis spectrophotometer at 48 hrs also suggests that the CdS nanoparticles synthesis process was completed at 24 hrs. The CdS nanoparticles are energetically synthesized at this phase. Similar observations are also noted in the report by Kalishwaralal et al. [19]. The long-term stability of the CdS nanoparticles solution is due to the presence of the proteins in the nanoparticles solution that bind to the surface of the nanoparticles and prevent aggregation [20]. In* K. pneumoniae*, the reduction of ZnSO_4_ metal ions to ZnS nanoparticles are started at 6 hrs and it is confirmed by the color change from yellow to light white color and the intensity of white color is gradually increased which reveals that the biomolecules reduced the ZnSO_4_ into ZnS nanoparticles as shown in Figures 1(c) and 1(d). The color changes depend upon the incubation time (6–24 hrs) and size and shape of the nanoparticles. The ZnS nanoparticles are formed in the reaction mixture due to the effect of active biomolecules of microorganism and the present report was correlated with the report of* R. sphaeroides* [21] and bacteriophage [22] synthesized ZnS nanoparticles. In* K. pneumoniae* cultures, the CdS and Zns nanoparticles are actively synthesized at 24 hrs. After 24 hrs, no color change was observed. Only the precipitation is observed at the bottom of the conical flask which indicates that the sulphide nanoparticles synthesis process was completed. The 24 hrs culture is considered as stationary phase of the biomass, active cells in the stationary phase of biomass.

#### 3.2.2. UV-Vis Spectrophotometer

**Figure 2:**
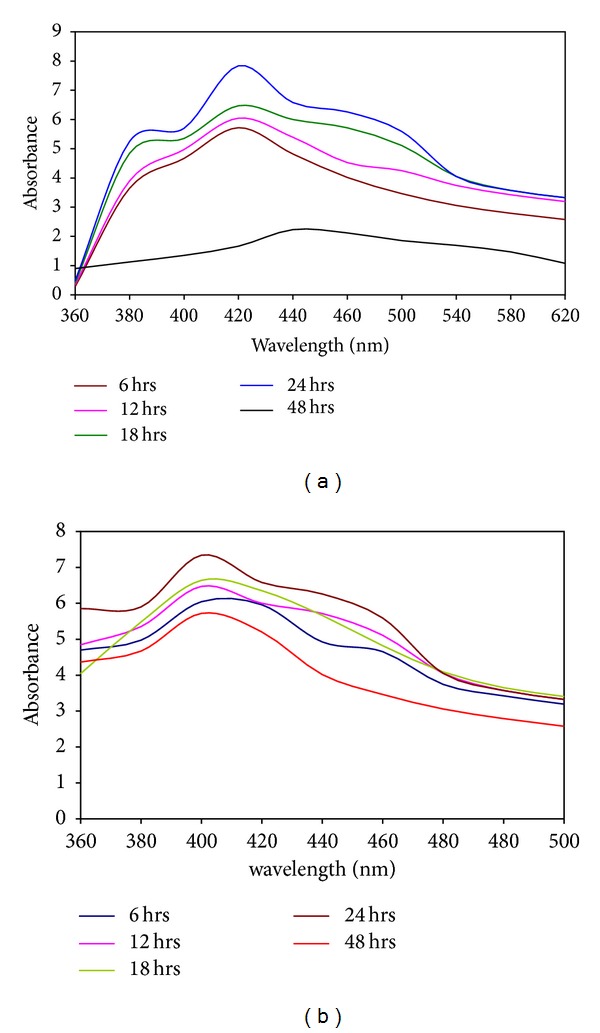
Absorption spectrum of nanoparticles synthesized by the culture supernatant of* K. pneumoniae and* SPR band at 420 nm for CdS (a) and 400 nm for ZnS (b).

Figures 2(a) and 2(b) exhibit the UV-Vis spectrophotometer of synthesized CdS and ZnS nanoparticles by using* K. pneumoniae *(MAA). The cadmium sulphate and zinc sulphate solutions were treated with biomass* K. pneumoniae *at different time intervals (6, 12, 24, and 48 h) assuming that different growth phase plays an important role in nanoparticles synthesis process. The broad peak was located between 380 and 420 nm for CdS and ZnS solutions. The absorbance of CdS NPs that was gradually increased from 6 to 24 hrs indicates gradual increase of nanoparticles synthesis. At 24 hrs, the surface plasmon resonance band was observed at 420 nm. The decreased absorbance at 48-hour incubation reveals that the reaction was completed at 24 hrs. The similar peak was observed for nanoparticle synthesized by using* K. pneumoniae* [23],* S. nematodiphila* [24, 25], and* R. paultris* [21]. Moreover, the plasmon bands are broadened with an absorption end in the longer wavelengths, which may be due to the size distribution of the particles [26]. Similarly, the ZnS nanoparticles synthesized extracellularly using* K. pneumoniae* had an absorption peak of 400 nm and the intensity of the peak was found increasing as the progress development of the reaction continues which explains an increase in the number of particles. Generally in biomass, ZnSO_4_ reduce ZnS nanoparticles and settle down at the bottom of the conical flask. As the size of the ZnS nanoparticles increases, the colors of the solution turn from yellow to white color with precipitation. After 48 hrs of incubation, the rate of nanoparticles formation was reduced. After 24 hrs, the absorbance was gradually decreased which indicates the ZnS nanoparticles synthesis process was completed. Appearance of this absorption shoulder together with bulge at 400 nm indicates the presence of nanocrystallites with different sizes. This observation is well supported by TEM analysis which shows the presence of different types of particles, respectively. Similarly, our results coincide with synthesized ZnS nanoparticles using the* R. sphaeroides* [27].

#### 3.2.3. XRD

**Figure 3:**
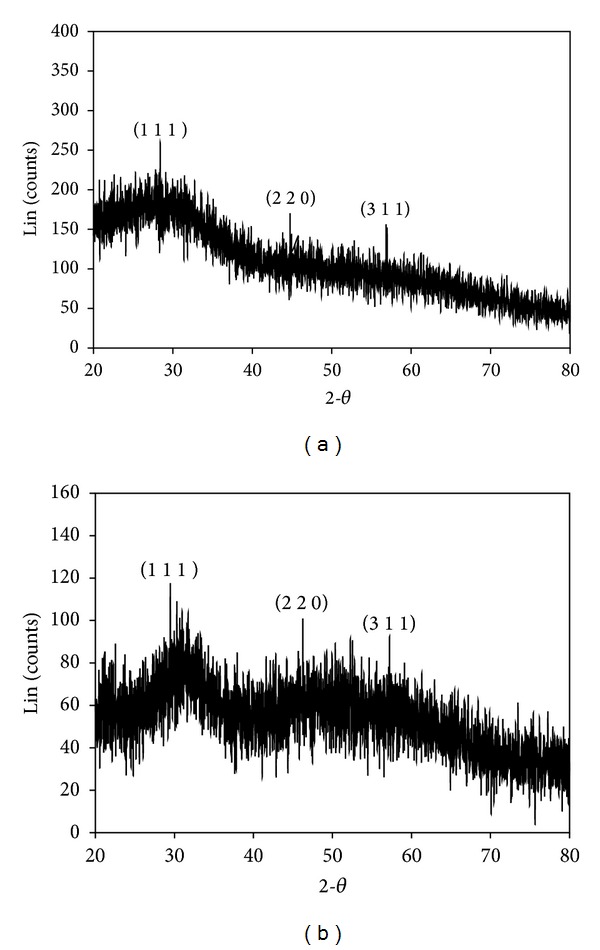
XRD pattern of sulfide nanoparticles synthesized by* K. pneumoniae*: (a) CdS and (b) ZnS.

X-ray diffraction profiles of synthesized cadmium sulphide and zinc sulphide nanoparticles by using* K. pneumoniae* were shown in Figures 3(a) and 3(b), respectively. The diffraction peak of CdS synthesized by* K. pneumoniae* was observed at 2*θ* values of 26.5°, 44.6°, and 52.8° which can be indexed to the (1 1 1), (2 2 0), and (3 1 1) planes of cubic crystalline CdS [21]. Likewise, the* K. pneumoniae* derived ZnS NPs exhibit the diffraction peaks at 29.23°, 46.05°, and 57.86° corresponding to the (1 1 1), (2 2 0), and (3 1 1) set planes which indicates that the ZnS are crystalline in nature. These peaks are matched with pure CdS and ZnS which were published by Joint Committee for Powder Diffraction standards (JCPDS File no. 454 for CdS and 566 for ZnS). The Bragg sharp peaks force has resulted due to the capping agent and stabilizing the nanoparticle. The average size was found to be 6.77 nm for CdS and 18 nm for ZnS, respectively, which was calculated by using full-width half maximum (FWHM) of the strongest peak in plane (1 1 1). Similar result was obtained by the chemical synthesis of ZnS nanoparticles, reported by Ni et al. [28]. The agglomeration of the neighbor growing crystallites helps to restrict the particle size in nanometer range. By controlling the deposition time, the particle size can be varied. This is due to the fact that the deposition time increases, number of atoms arriving on the substrate surface (atom) also increases, and these atoms will migrate on the surface with some activation energy [29]. The result was correlated with the report of Bai et al. [21] and Kho et al. [30].

#### 3.2.4. TEM

**Figure 4:**
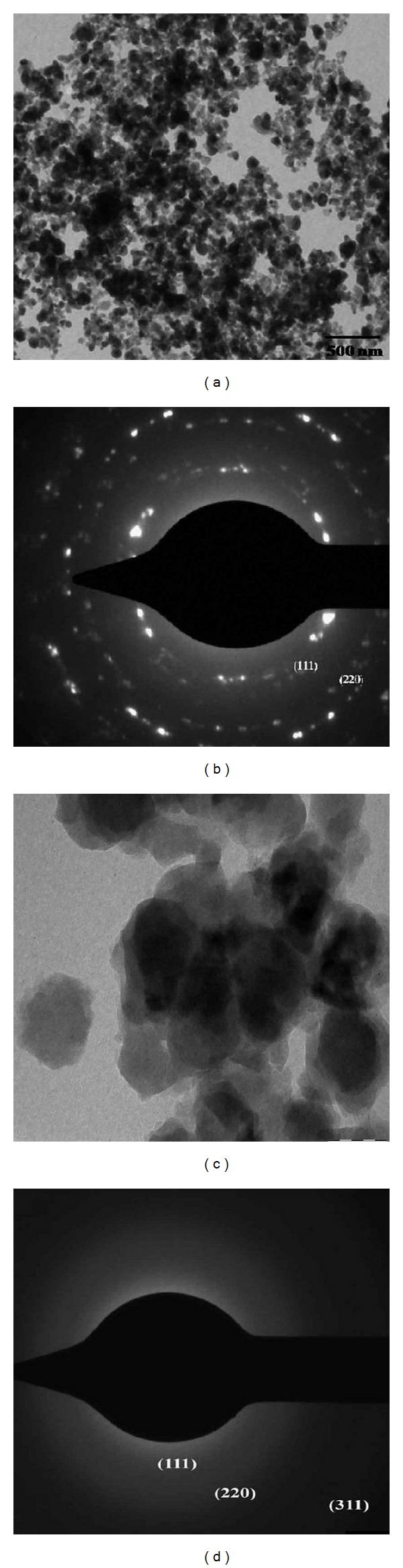
TEM image of synthesized sulfide nanoparticle by* K. pneumoniae*: (a) CdS and (b) ZnS, and SAED pattern of sulfide nanoparticle: (c) CdS and (d) ZnS.

The TEM image of* K. pneumoniae* derived CdS NPs and ZnS NPS is shown in Figures 4(a) and 4(c), respectively. TEM technique is used to visualize the shape and size of the sulfide nanoparticles showing ploydispersed small spherical to large spherical shape. The sulfide nanoparticles are polydispersed, mostly spherical in shape with few numbers of aggregates. The particles are embedded in the matrix indicating that the samples are composed of a large number of well dispersed nanoparticles with irregular size and shape and they are grouped to form clusters. Previously, Sanghi and Verma [25] have reported the synthesis of CdS nanoparticles in a cluster form which was surrounded by a thin outer not so dense layer that might be a protein. The average size of the* K. pneumoniae* derived CdS nanoparticles is 10 to 25 nm (Figure 4(a)) and 65 nm for ZnS (Figure 4(c)). The present report was correlated with the report of Ahmad et al. [31] where they have synthesized spherical shaped CdS nanoparticles by using fungus* Fusarium oxysporum*. The reason for the increase of the particle size with the bacterial growth from the exponential phase to the stationary phase is probably due to “nucleation effect,” where small particles agglomerate to form the larger particle [32]. According to Bai et al. [27] there was formation of spherical shape due to the aggregation in particles as well as the settlement at the bottom of the flask. This image matched the report of ZnS synthesized by using* R. sphaeroides* [21].

#### 3.2.5. SAED Pattern

The SAED pattern of the single particle shows the sharp diffraction spots which clearly suggest that the particles are in single crystal quality and the plane could be indexed to the fcc of CdS and ZnS shown in Figures 4(b) and 4(d). It consists of three sharp concentric rings with distinctive spots on the rings. The sharp rings revealed the polycrystalline nature of the nanoparticles. These patterns are obtained from the lattice reflections of (1 1 1), (2 2 0), and (3 1 1) with the same interplanner spacing as those obtained from XRD. Related three concentric rings are observed in the zinc sulfide nanoparticle which indicates that the particles are of crystalline nature [28].

#### 3.2.6. FTIR

**Figure 5:**
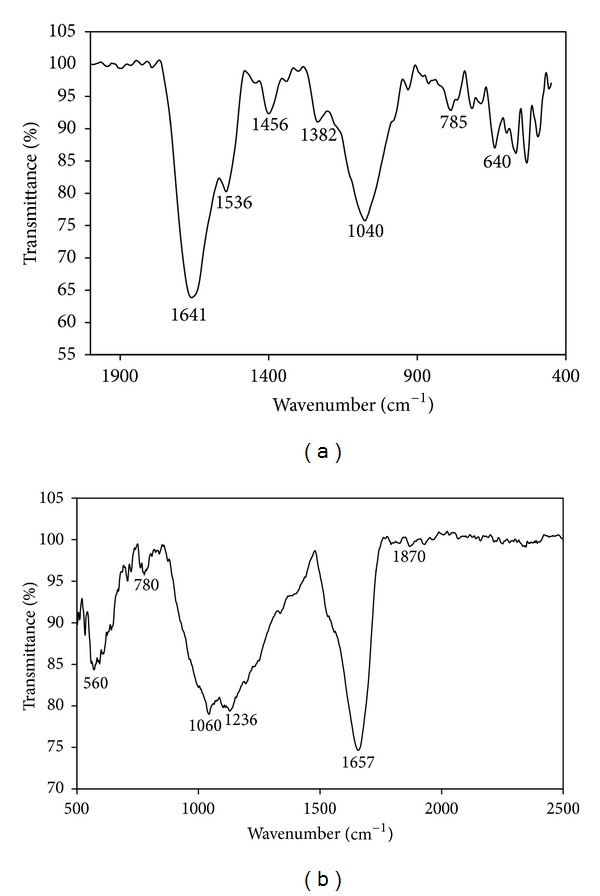
FTIR spectra recorded from powder of sulfide nanoparticles synthesized using* K. pneumoniae*: (a) CdS and (b) ZnS.

FTIR measurements were carried out to identify the possible biomolecules responsible for the reduction of sulfate (cadmium and zinc) and capping of the bioreduced sulphide nanoparticles synthesized by* K. pneumoniae*. A number of vibration bands can be seen in the region 4000–400 cm^−1^. Absorption spectra observed in the region of 2000–400 cm^−1^ are 1641 cm^−1^, 1536 cm^−1^, 1382 cm^−1^, 1040 cm^−1^, 785 cm^−1^, and 640 cm^−1^ for CdS shown in Figure 5(a) and 560 cm^−1^, 780 cm^−1^, 1060 cm^−1^, 1236 cm^−1^, 1657 cm^−1^, and 1870 cm^−1^ for ZnS shown in Figure 5(b). The absorption peaks located at around 1641 cm^−1^ can be assigned to the N–H stretching vibrations due to the primary amines and secondary amines linkages of proteins and amino acid residues in polypeptides, respectively. Sanghi and Verma [25] have reported that the proteins can bind to CdS nanoparticles either through free amine groups or cysteine residues vital role in the protein. The current report also explained that cysteine residues may play a vital role in the formation of CdS nanoparticle. The strong peak observed at 1657 cm^−1^ corresponds to the carbonyl stretching vibrations amide I linkages of protein in ZnS nanoparticles [33]. Proteins can bind the nanoparticle through free amine groups and cysteine residues of proteins [34–36]. The bands seen at 1536 cm^−1^ were assigned to the C=C bending vibrations due to aromatics (removed, resp.). The bands can exist at 1040 cm^−1^, 1060 cm^−1^, 1382 cm^−1^, and 1236 cm^−1^ indicates C–N stretching vibrations due to aliphatic and aromatic groups, respectively. Previously, Bai et al. [21] reported that the aliphatic and aromatic groups were involved in the synthesis of CdS nanoparticle by using* R. palustris*. The small peak at 785 cm^−1^ and 780 cm^−1^ due to N–H stretching vibrations indicates the presence of primary and secondary amines of proteins. The bands seen at 640 cm^−1^ and 560 cm^−1^ are identified as alkyl halides and arise due to C–Cl stretching. IR study confirms the presence of amide groups and aliphatic residues of proteins have the stronger ability to bind with metal, so that the protein is most possibly covered by metal nanoparticle [37]. According to Philip [38], the presence of –COOH due to C=O stretching vibrations of protein on the surface of the nanoparticle is proposed. These protein molecules perform as surface coating molecules which keep the particles away from the internal agglomeration.

#### 3.2.7. Mechanism on Biosynthesis of CdS and ZnS Nanoparticles

**Figure 6:**
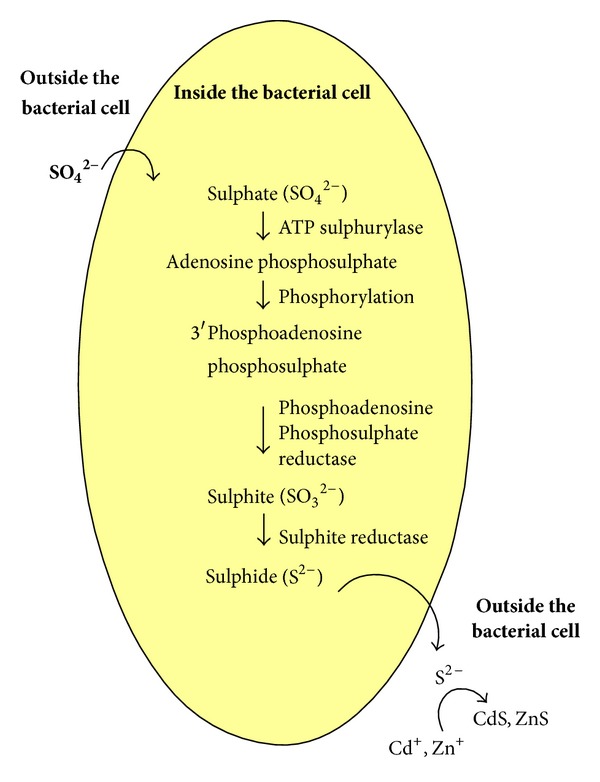
Probable pathway of biosynthesis and stability of biofunctionalized sulfide nanoparticle.

The present study reports the biological synthesis of sulphide nanoparticles like CdS and ZnS using the bacteria* K. pneumoniae*. Aiking et al. (1982) have been reported that the* Klebsiella aerogens* has undergone two different detoxification processes against sulphate and phosphate such as metal sulphide and metal phosphate formation. Additionally, they suggested that the cadmium ions could be detoxified by the formation of extracellular cadmium sulphide particles [38–40]. Similarly, herein,* K. pneumoniae* detoxifies the cadmium sulphate and zinc sulphate and synthesizes the nanosized CdS and ZnS particles, respectively. In synthesis process (Figure 6), first, the sulphate ions are taken in (SO_4_^2−^) from the extracellular environment (nutrient broth medium) and reduced to adenosine phosphosulphate with the support of the enzyme ATP sulfurylase [40]. The adenosine phosphosulphate further phosphorylated to form 3′phosphoadenosine phosphosulphate. The 3′phosphoadenosine phosphosulphate is reduced to form sulphite ions (SO_3_^2−^) with the assistance of phosphoadenosine phosphosulphate reductase [39]. Then, the sulphite ions are reduced to sulphide ions (S^2−^) with the help of sulphite reductase. The reduced sulphide ions are coupled with inorganic metal ions cadmium and zinc in extracellular environment and resulted in the formation of cadmium sulphide and zinc sulphide nanoparticles [40, 41]. Likewise sulphate ions, the selenium ions are also reduced to selenide ions through enzymatic reaction. Previously, Li et al. (2007) have demonstrated the reduction of selenium ions with the assistance of 30 kDa protein molecules present in the extract of* Capsicum annuum* [42]. The presence of phytochemicals and secondary metabolites in the plant extracts also have the capability to synthesis inorganic nanoparticles [43].

#### 3.2.8. Fluorescence Emission of CdS and ZnS Nanoparticles

**Figure 7:**
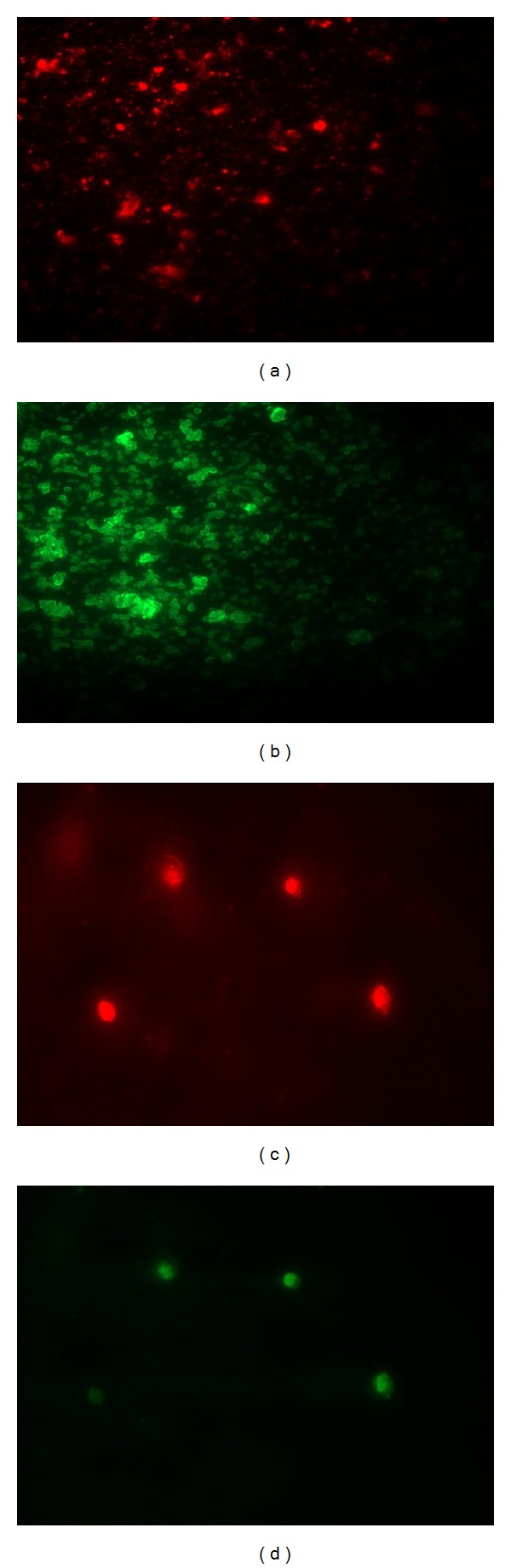
Fluorescence image of (a) CdS nanoparticles and (b) ZnS nanoparticles synthesized from* K. pneumoniae.*

CdS and ZnS also known as colloidal semiconductor nanocrystals are novel inorganic fluorescence labeling agents [44, 45]. The results obtained clearly exhibit the fluorescent intensity of CdS and ZnS nanoparticles. Figures 7(a) and 7(b) show the characteristic “green” and “red” fluorescence image of CdS nanoparticle in aqueous colloidal solution excited by laser source at 442 nm. Figures 7(c) and 7(d) represent the image of synthesized ZnS nanoparticles with a bright green fluorescence when exposed to ultraviolet radiation.The results have given support for considering this novel system based on CdS to be potentially used in biolabeling applications. Mansur et al. [46] suggest that synthesis of CdS and ZnS nanocrystals represents hybrid inorganic-organic materials that can be both fabricated and functionalized with biomolecules in a relative facile synthetic route. In the present study, the synthesis of semiconductor nanoparticles opens a wide window of possibilities in biomedical research and applications for producing water soluble inorganic fluorophores based on quantum dots.

#### 3.2.9. Antimicrobial Activity of Sulphide Nanoparticles

The antimicrobial activity of* K. pneumoniae* nanoparticles was investigated against pathogenic bacteria, such as* Staphylococcus aureus*,* Lactobacillus *sp.,* Streptococcus *sp., and* Candida albicans*. The diameter of zone of inhibition (ZOI) was measured around the well with nanoparticles against the test strains. The ZOI was expressed in millimeter (mm) for the oral pathogenic bacteria. The various concentrations of sulphide nanoparticle were 100 *μ*L, 200 *μ*L, and 300 *μ*L.  Table 1 shows the synthesis of CdS nanoparticle by using* K. pneumoniae.* The highest antimicrobial activity was experimental against bacteria* Staphylococcus aureus* (25 ± 0.33) and* Lactobacillus* sp. (23 ± 0.4). This clearly demonstrates that the antibacterial activity is simply due to CdS nanoparticle impregnated and surrounded by the bacterial cell. The antibacterial activity was found to be quite strong and good.  Table 2 shows the synthesis of ZnS nanoparticle by using* K. pneumoniae*. The bacterial growth rate of* Lactobacillus* sp. (23.66 ± 0.69),* Staphylococcus aureus* (25.33 ± 0.35),* Candida albicans* (19 ± 0.89), and* Streptococcus* sp. (21 ± 0.55) was decreased while increasing the concentration of zinc sulphide nanoparticles. The formation of zone around the ZnS nanoparticles integrated well clearly motivated the antibacterial property of ZnS nanoparticles.

Regarding the mechanism of antibacterial activity of nanoparticles, gram negative bacteria showed more inhibition zone than the gram positive bacteria due to the cell wall nature of the bacteria. The gram positive bacteria formation of the cell wall is collected of deep layer of membrane, consisting of linear polysaccharide chains, and the gram negative bacteria possess the slender layer of membrane. Shukla et al. [47] reported that nanoparticles discharge the ions, which react with the thiol groups in the proteins present on the bacterial cell membrane. Such proteins outcropping during the bacterial cell surface allows carrying nutrients through the cell membrane. Vanaja et al. [48] reported that gram positive bacteria have thick and chemically complex peptidoglycan in the cell wall, so nanoparticles are not easily entered into the cell. But the gram negative bacteria have thin simple multilayered lipid materials in the cell wall, so the nanoparticles did easily enter into bacterial cells and then showed an inhibition zone higher than the gram positive bacteria. The antibacterial effects and stabilized cadmium oxide nanoparticles can be helpful in the infectious disease treatment caused by* E. coli* [49]. Rajeshkumar et al. [50] reported that microbes transmit a positive charge. This creates an “electromagnet” attraction between the microorganisms and treats the cell outer membrane. The present study demonstrates that CdS and ZnS nanoparticles have bactericidal activity against the entire test organism. Since this is easily available in the nation and also is used in hospital for biomedical agent, the energetic nanocompound from this can be prepared and used effectively for preventing the growth of the oral pathogens.

#### 3.2.10. Semiconductor Nanocrystals Control the Oral Pathogens

The antifungal, antibacterial, and antiviral actions of CdS and ZnS compounds have been expansively investigated with assessment of other metals. The use of semiconductor nanoparticles has been well thought of for a range of biomedical applications, including, within the dental field, an antibacterial root in dental resin composites. The use of CdS and ZnS nanoparticles, as a substitute for elemental Cd, Zn, and S or complex CdS and ZnS nanoparticles, has been characterized and investigated with respect to their possible antimicrobial applications [51]. Biosynthesis of nanoparticles is being investigated for a variety of potential applications, for example, assimilation to denture materials [52] and orthodontic adhesives [53]. The present work is greatly used under* in vitro* condition, because the biocompatibility of nanoparticles is not completely addressed so far. Biofilm expansion is well known to contribute to minor caries and the breakdown of resin-based dental composites [54]. Zinc nanoparticles have undergone* in vitro* testing in biofilm culture experiment system. Zinc nanoparticles blended into a multiplicity of composites were shown to considerably inhibit* Staphylococcus sobrinus* biofilm increase at concentrations [55]. Hydroxyapatite material accessible in nanophase and nanocrystalline could be used as antimicrobial coating agent to reduce the possibility of bacterial migration [56].

## 4. Conclusion

In this present investigation, we newly reported the production of sulphide nanoparticles by using the biomass of* K. pneumoniae*. The formation of SPR band at 420 nm and 400 nm indicates the presence of CdS and ZnS nanoparticle, respectively, which are synthesized by culture supernatant of* K. pneumoniae*. The morphological (TEM and SAED) and structural (XRD) analysis including spectroscopic techniques (UV-Vis spectrophotometer and FTIR) confirmed that the bacteria might play an important role in the stabilization of CdS and ZnS nanoparticles. Green synthesized sulphide nanoparticles which had the bactericidal and fungicidal activity against* Streptococcus *sp.,* Staphylococcus aureus*,* Lactobacillus *sp., and* Candida albicans*, respectively, was successfully demonstrated by disc diffusion method with ZOI on the agar plate. The application of semiconductor sulphide nanoparticles to organize biofilm development surrounded by the oral cavity, as a function of their bactericidal activity and delivery capabilities, is valuable and of serious consideration. This green chemistry approaches is used to alter biocompatibility of nanoparticles and more beneficial for budding technology researcher to manufacture the nanoparticles using surface coatings and dental devices. Moreover, this process is eco-friendly and nontoxic, and handlings of oral pathogens are also biocompatible.

## Figures and Tables

**Table 1:** Zone of CdS nanoparticles against oral pathogenic microorganisms.

Concentration of cadmium sulfide nanoparticles	Zone of inhibition (mm in diameter)			
	*Streptococcus* sp.	*S. aureus *	*Lactobacillus* sp.	*C. albicans *
100 *μ*L	15.66 ± 0.40	16.66 ± 0.42	10.66 ± 0.95	11.66 ± 0.44
200 *μ*L	18.33 ± 0.46	19.33 ± 0.5	12.66 ± 0.27	15.33 ± 0.32
300 *μ*L	26.33 ± 0.63	25.33 ± 6.42	15 ± 3.27	17.66 ± 4.32

±Standard deviation.

**Table 2:** Antimicrobial activity of ZnS nanoparticles against oral pathogens.

Concentration of zinc sulfide nanoparticles	Zone of inhibition (mm in diameter)			
	*Streptococcus* sp.	*S. aureus *	*Lactobacillus* sp.	*C. albicans *
100 *μ*L	15.33 ± 0.41	15.33 ± 0.32	9.66 ± 0.47	12.33 ± 0.43
150 *μ*L	17.66 ± 0.44	18.33 ± 0.47	14.33 ± 0.38	16 ± 0.22
200 *μ*L	24.33 ± 0.18	25.66 ± 0.60	16.66 ± 0.42	18.66 ± 0.65

±Standard deviation.
